# Straighter low lumbar curvature in isthmic spondylolisthesis at L4

**DOI:** 10.1186/s12891-020-03519-4

**Published:** 2020-07-22

**Authors:** Shaoli Zheng, Zhaoming Zhong, Qingan Zhu, Zongze Li, Siyuan Zhu, Xinqiang Yao, Shuai Zheng, Congrui Liao, Yongjian Zhu, Jianting Chen

**Affiliations:** Department of Spinal Surgery, Nanfang Hospital, Southern Medical University, 1838 North Guangzhou Avenue, Guangzhou, China

**Keywords:** Isthmic spondylolisthesis, Sagittal lumbo-pelvic alignment, Lower lumbar lordosis, L5 incidence, Roussouly type

## Abstract

**Background:**

This study was conducted to compare differences in imaging features and clinical symptoms between patients with single-level isthmic spondylolisthesis (IS) at L4 and at L5 and to investigate the correlation between imaging and clinical parameters.

**Methods:**

This cross-sectional study evaluated patients with single-level IS who were enrolled between June 2011 and June 2018. A total of 139 patients, 44 in the L4 IS group and 95 in the L5 IS group, met the study criteria and were included. Imaging and clinical parameters obtained from the two groups were compared and analyzed.

**Results:**

Patients in the L4 IS group had smaller lower lumbar lordosis (LLL) (27.1 ± 8.2 vs. 30.9 ± 9.3, *P* = 0.021) and were of older age (58.5 ± 8.7 vs. 52.8 ± 10.1, *P* < 0.01) than those in the L5 IS group. As per the Roussouly classification system, most patients with L4 IS were classified as Type 2 (43.2%), whereas most patients with L5 IS fell under Type 3 (44.2%). In the L5 IS group, pelvic incidence (PI), pelvic tilt (PT), sacral slope (SS), lumbar lordosis (LL), and L5 incidence (L5I) were positively associated with slippage rate (SR), but the lumbosacral angle (LSA) was negatively associated with SR (*P* < 0.01). In the L4 IS group, only L5I showed a positive association with SR (P < 0.01). More significant associations were found among sagittal lumbo-pelvic parameters in the L5 IS group, but none were found between SR and Oswestry Disability Index (ODI) in either group.

**Conclusions:**

When compared with patients with L5 IS, patients with L4 IS were of older age and had straighter low lumbar curvature when they were obviously symptomatic. PI was an important parameter for patients with L5 IS while for those with L4 IS, L5I deserved more attention for its significantly positive correlation with the degree of slippage.

## Background

Spondylolysis is characterized as a defect in the bilateral pars interarticularis of the vertebral arch and can be caused by genetic factors, trauma, and repetitive exercise [1]. Isthmic spondylolisthesis (IS), a complication of spondylolysis, is defined by the anterior slippage of one vertebra with a defect in bilateral pars interarticularis over the next caudal one [2]. The main clinical symptoms of IS are back pain, sciatica and/or intermittent claudication. Spondylolisthesis occurs in 50–81% of patients with bilateral spondylolysis [3]. IS occurs most commonly at L5 and second most commonly at L4, given that > 70% of spondylolytic lesions occur at L5 and approximately 15% at L4 [4].

Grobler et al. [5] performed biomechanical experiments on six specimens to confirm that the lesion segment was more unstable when spondylolytic lesions occurred at L4 than at L5. Through an observational study on 665 skeletal lumbar spines, McCunniff et al. [6] found that patients with spondylolysis at L4 also displayed a greater degree of degeneration of the disc below the level of the associated isthmic defect than those with spondylolysis at L5, suggesting that there was a greater degree of degenerative disc disease and clinical symptoms among people with spondylolysis at L4. Most patients with spondylolysis progressed to IS when they had obvious clinical symptoms [7]. However, the differences between single level IS at L4 and L5 have been poorly characterized in clinical studies. In addition, a number of researchers have reported that sagittal lumbo-pelvic alignment has a significant influence on the clinical symptoms and progression of IS [8–10], but most of these studies only focus on IS at L5 or ignored the differences between IS at L4 and L5.

The current study mainly compared the differences in ODI, sagittal lumbo-pelvic alignment, disc degeneration grade and slippage rate of the lesion segment between single-level IS at L4 and L5. We also analyzed the correlations between imaging measurements and ODI for IS at L4 and L5 and observed their differences.

## Methods

### Patients

This study was retrospectively conducted on patients who were hospitalized for L4 or L5 IS between June 2011 and June 2018. Inclusion criteria of the study were as follows: (1) age 18 years or older at the time of hospitalization; (2) definite diagnosis of single-level L4 or L5 IS (anterior slippage of the vertebral body in more than 5% of cases associated with spondylolysis of the pars interarticularis); (3) complete imaging data including standing lateral lumbar X-ray films and lumbar magnetic resonance imaging (MRI). Exclusion criteria were as follows: (1) a history of previous spinal surgery, trauma or infection; (2) accompanying with spinal tumor, multilevel spondylolysis, unilateral pars defect, scoliosis, or lumbosacral transitional vertebra.

Demographic data of the patients, such as age, sex, body mass index (BMI), and any existing comorbidities were recorded. Patients’ complications including Diabetes and Osteoporosis were collected from Electronic Medical Record System of the hospital. To assess the severity of clinical symptoms, all patients completed the Oswestry Disability Index (ODI) questionnaire in the hospital before undergoing surgical or other treatments.

### Imaging measurements

**Fig. 1:**
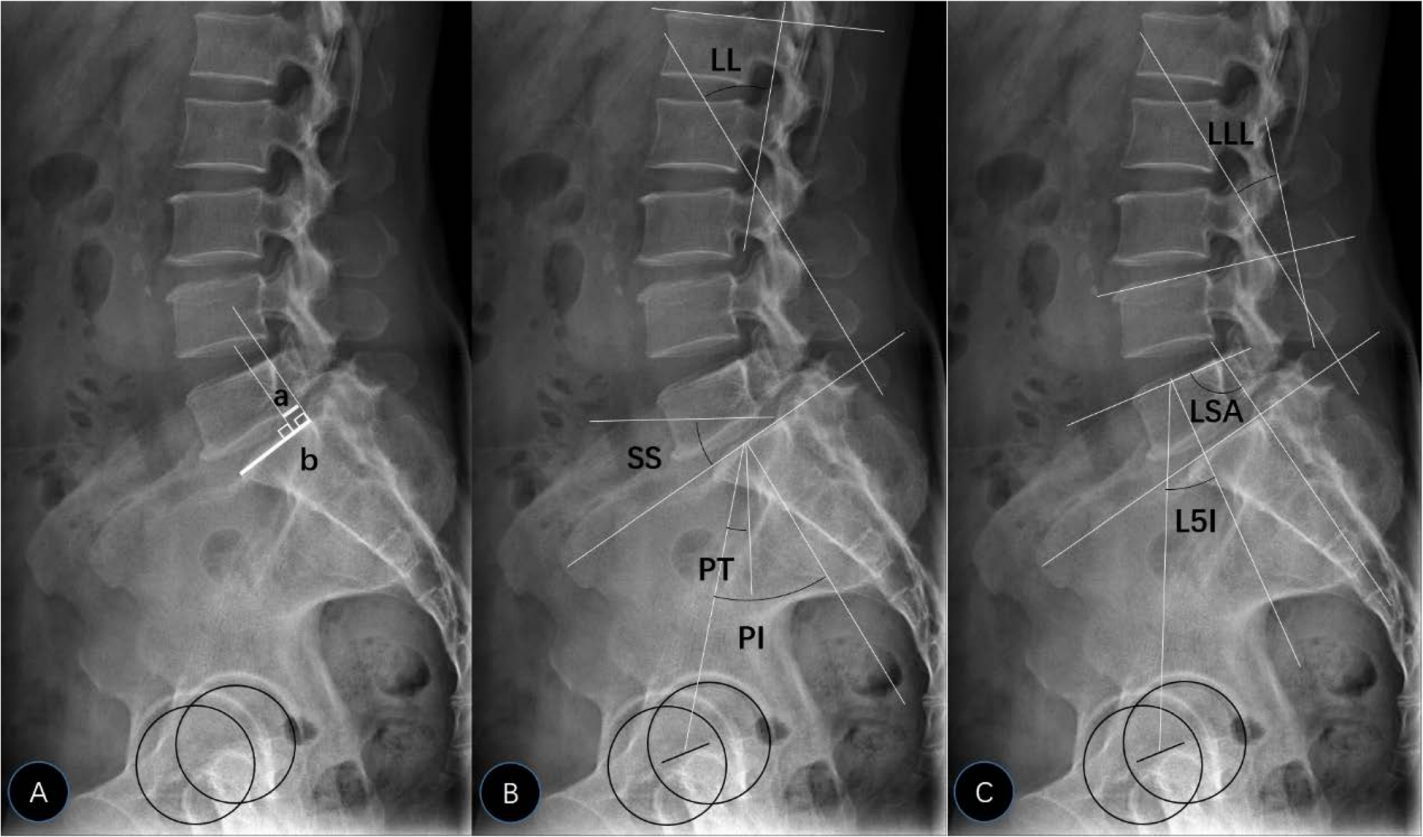
Measuring method of parameters of lateral X-ray film. **a** Measuring method of SR: SR = a/b*100%. (a: distance between the vertical line of superior endplate of the lower vertebra through posterior end of inferior endplate of upper vertebra and the vertical line of superior endplate of the lower vertebra through posterior end of superior endplate of lower vertebra. b: length of superior endplate of lower vertebra.) **b** Measuring method of PI, PT, SS, and LL. **c** Measuring method of LLL, LSA, and L5 I

Each patient underwent a standing lateral radiography of the lumbo-pelvic region including the first lumbar and bilateral femoral heads as per the standardized protocol. Lumbar MRI was performed to assess the degree of disc degeneration. The disc we assessed in our study was below the level of the associated isthmic defect. The disc degeneration grade (DDG) was evaluated using the modified Pfirrmann grading system on T2-weighted sagittal MRI [11]. As shown in Fig. 1, the parameters of sagittal lumbo-pelvic alignment in the current study included pelvic incidence (PI), pelvic tilt (PT), sacral slope (SS), lumbar lordosis (LL), lower lumbar lordosis (LLL), L5 incidence (L5I), and lumbosacral angle (LSA).

PI was measured as the angle between the perpendicular bisector of the S1 endplate and the line connecting the midpoints of the S1 upper endplate and center of the femoral heads. PT was measured as the angle between the line connecting midpoints of the S1 upper endplate and the center of the femoral heads and the vertical line. SS was measured as the angle between the tangent of the S1 endplate and the horizontal line. LL was measured as the angle between the superior endplate of L1 and the superior endplate of S1. LLL was measured as the angle between the superior endplate of L4 and the superior endplate of S1 [12]. L5I was measured as the angle subtended by the line drawn from the center of the femoral heads to the midpoint of the upper L5 end plate and the line perpendicular to the upper L5 end plate [13]. LSA was measured as the angle between the superior endplate of L5 and the posterior cortex of S1 [14]. Slippage rate (SR) was defined as per the technique recommended by Taillard [15]. Sagittal lumbo-pelvic alignments in both groups were classified into four types defined by Roussouly et al. [16] as follows: (1) Type 1 was defined as SS < 35° with the apex of lordosis located at the center of the vertebral body L5. (2) Type 2 was defined as SS < 35° with the apex of LL located at the base of the vertebral body L4. (3) Type 3 was defined as SS between 35° and 45° with the apex of LL located at the center of the vertebral body L4. (4) Type 4 was defined as SS > 45°, with the apex of LL at the base of vertebral body L3 or higher. Roussouly types are estimated based on LL and SS. All imaging measurements in this study were measured on an institutional picture archiving and communication system (PACS) by two spine surgeons and inter-observer reliability was tested.

### Statistical analysis

Student’s t-test and chi-square test were performed for comparison of continuous or categorical variables between the L4 and L5 IS groups. Analysis of covariance (ANCOVA) was performed for age-matched analysis. Fisher’s exact test was performed to compare the distribution of the Roussouly type. Pearson’ correlation analysis was performed to analyze the correlation between imaging measurements and ODI for each group. For testing interobserver reliability, continuous variables were assessed using the intraclass correlation coefficient, and categorical variables were analyzed using kappa values. All analyses were performed using the Statistical Package for the Social Sciences (SPSS) software version 21.0, with *P* < 0.05 considered to indicate statistical significance.

## Results

A total of 139 patients, 44 cases of L4 IS, and 95 cases of L5 IS, were included in our study. The inter-observer reliability ranged from 0.83 to 0.92, indicating good agreement between the two spine surgeons who performed all measurements.

Basic characteristics of the included patients and comparison of the parameters between two groups are listed in Table 1. There were no significant between-group differences in sex distribution, BMI, or proportion of patients with diabetes or osteoporosis (*P* > 0.05). However, the average age of the L4 IS group was significantly higher than that of the L5 IS group (58.5 ± 8.7 vs. 52.8 ± 10.1, *P* < 0.01). There was no difference between the two groups in terms of PI, PT, LL, L5I, LSK, SR, and DDG. LLL in the L4 IS group was significantly smaller than that in the L5 IS group (27.1 ± 8.2 vs. 30.9 ± 9.3, *P* = 0.021). To further characterize the influence of age in the two groups, an age-matched analysis was performed and the results were similar to those in the previous analysis (Table 2). LLL in the L4 IS group was also significantly smaller (26.7 ± 1.4 vs. 31.0 ± 0.9, *P* = 0.012). The distribution of different Roussouly types in patients with L4 and L5 IS is listed in Table 3. Among patients in the L4 IS group, the most common was Type 2 (43.2%), and there were no patients in the Type 1 category. However, in the L5 IS group, most patients were classified as Type 3 (44.2%). The difference in the frequency of Roussouly types between the two groups was significant (*P* = 0.001).

**Table 1:** Comparison of parameters between the L4 IS group and the L5 IS group

Parameter	Isthmus Spondylolisthesis		Test Result	P Value
	L4	L5		
No. of patients	44	95	–	–
Age in yrs	58.5 ± 8.7	52.8 ± 10.1	t = −3.232	0.002 *
Sex (male/female)	15/29	29/66	χ2 = 0.177	0.674
BMI (kg/m^2^)	25.1 ± 4.3	24.6 ± 3.5	t = −0.640	0.523
Diabetes (yes/no)	4/40	15/80	χ2 = 1.143	0.285
Osteoporosis (yes/no)	3/41	7/88	χ2 = 0.014	0.907
PI(°)	58.4 ± 9.5	59.3 ± 12.4	t = 0.409	0.683
PT(°)	22.6 ± 9.6	20.0 ± 8.8	t = −1.616	0.108
SS(°)	36.1 ± 9.8	39.7 ± 10.6	t = 1.892	0.061
LL(°)	45.5 ± 13.2	48.4 ± 13.5	t = 1.190	0.236
LLL(°)	27.1 ± 8.2	30.9 ± 9.3	t = 2.326	0.021*
L5I(°)	29.9 ± 8.0	30.7 ± 13.0	t = 0.285	0.776
LSK(°)	106.8 ± 16.3	104.8 ± 14.5	t = −0.726	0.496
SR(%)	25.6 ± 7.3	25.1 ± 11.6	t = − 0.309	0.758
DDG	6.4 ± 1.5	5.9 ± 1.6	t = −1.855	0.066
ODI	50.1 ± 15.0	48.0 ± 15.1	t = −0.624	0.534

Data are presented as number of patients or mean ± SD. BMI Body mass index; PI Pelvic incidence; PT Pelvic tilt; SS Sacral slope; LL Lumbar lordosis; LLL Lower lumbar lordosis; L5 I L5 incidence; LSA Lumbosacral angle; SR Slippage rate; DDG Disc degeneration grade; ODI Oswestry Disability Index

*Statistically significant (*P* < 0.05)

**Table 2:** Comparison of parameters between the L4 IS group and the L5 IS group (After age-matched analysis)

Parameter	Isthmus Spondylolisthesis		Test Result	P Value
	L4	L5		
PI(°)	58.2 ± 1.8	59.4 ± 1.2	F = 0.3	0.563
PT(°)	22.8 ± 1.4	20.0 ± 0.9	F = 2.6	0.107
SS(°)	35.8 ± 1.6	39.8 ± 1.1	F = 4.2	0.052
LL(°)	44.7 ± 2.1	48.8 ± 1.4	F = 2.6	0.112
LLL(°)	26.7 ± 1.4	31.0 ± 0.9	F = 6.5	0.012*
L5I(°)	30.5 ± 2.2	30.4 ± 1.5	F = 0	0.945
LSK(°)	106.6 ± 2.3	104.8 ± 1.6	F = 0.4	0.574
SR(%)	25.5 ± 1.6	25.1 ± 1.1	F = 0.1	0.83
DDG	6.3 ± 0.2	5.9 ± 0.2	F = 2.4	0.126
ODI	48.8 ± 2.8	48.6 ± 1.8	F = 0	0.942

Data are presented as number of patients or mean ± SD. BMI Body mass index; PI Pelvic incidence; PT Pelvic tilt; SS Sacral slope; LL Lumbar lordosis; LLL Lower lumbar lordosis; L5 I L5 incidence; LSA Lumbosacral angle; SR Slippage rate; DDG Disc degeneration grade; ODI Oswestry Disability Index

*Statistically significant (*P* < 0.05)

**Table 3:** Distributions of Roussouly type among patients with L4 IS and L5 IS

	Isthmus Spondylolisthesis		P Value
	L4	L5	
Roussouly type			0.001*
1	0(0%)	11(11.6%)	
2	19(43.2%)	15(15.8%)	
3	15(34.1%)	42(44.2%)	
4	10(22.7%)	27(28.4%)	

* Statistically significant (*P* < 0.05)

Pearson correlations were conducted among imaging and clinical parameters for each group. The results are shown in Tables 4 and 5. In the L5 IS group, PI, PT, SS, LL and L5I were positively associated with SR, but LSA was negatively associated with SR (*P* < 0.05). In the L4 IS group, only L5I showed a positive association with SR (P < 0.05). In terms of the relationship between sagittal lumbo-pelvic alignment and clinical symptoms, SS and L5I was correlated with ODI in the L5 IS group (r = − 0.24, P < 0.05; r = − 0.32, P < 0.05), while no parameters were seen to be correlated with ODI in the L4 IS group. Upon comparing Tables 4 and 5, more significant correlations were found among parameters of sagittal lumbo-pelvic alignment in L5 IS group. We also found no significant correlation between SR and ODI in either the L4 IS or L5 IS group.

**Table 4:** Correlations between clinical and imaging measurements in the L4 IS group

	ODI	SR	PI	PT	SS	LL	LLL	L5I	LSK
**ODI**	**1**								
**SR**	**−0.14**	**1**							
**PI**	**−0.11**	**0.26**	**1**						
**PT**	**0.06**	**0.09**	**0.46**^a^	**1**					
**SS**	**−0.19**	**0.18**	**0.49**^a^	**−0.54**^a^	**1**				
**LL**	**−0.17**	**0.27**	**0.51**^a^	**−0.40**^a^	**0.87**^a^	**1**			
**LLL**	**−0.14**	**0.28**	**0.30**	**−0.24**	**0.53**^a^	**0.53**^a^	**1**		
**L5I**	**0.11**	**0.61**^a^	**0.64**^a^	**0.45**^a^	**0.21**	**0.25**	**−0.15**	**1**	
**LSA**	**−0.18**	**− 0.04**	**0.12**	**0.16**	**0.07**	**0.09**	**0.05**	**−0.21**	**1**

PI Pelvic incidence; PT Pelvic tilt; SS Sacral slope; LL Lumbar lordosis; LLL Lower lumbar lordosis; L5 I L5 incidence; LSA Lumbosacral angle; SR Slippage rate; ODI Oswestry Disability Index

^a^Correlations are significant at the 0.05 level (2-tailed)

**Table 5:** Correlations between clinical and imaging measurements in the L5 IS group

	ODI	SR	PI	PT	SS	LL	LLL	L5I	LSK
**ODI**	**1**								
**SR**	**−0.24**	**1**							
**PI**	**−0.24**	**0.52**^a^	**1**						
**PT**	**−0.74**	**0.33**^a^	**0.51**^a^	**1**					
**SS**	**−0.24**^a^	**0.33**^a^	**0.70**^a^	**−0.24**^a^	**1**				
**LL**	**−0.20**	**0.35**^a^	**0.65**^a^	**−0.17**	**0.88**^a^	**1**			
**LLL**	**−0.13**	**−0.20**	**0.26**^a^	**−0.20**	**0.45**^a^	**0.45**^a^	**1**		
**L5I**	**−0.32**^a^	**0.65**^a^	**0.78**^a^	**0.58**^a^	**0.42**^a^	**0.44**^a^	**−0.28**^a^	**1**	
**LSA**	**0.15**	**−0.42**^a^	**−0.28**^a^	**− 0.25**^a^	**−0.10**	**− 0.09**	**0.27**^a^	**− 0.51**^a^	**1**

PI Pelvic incidence; PT Pelvic tilt; SS Sacral slope; LL Lumbar lordosis; LLL Lower lumbar lordosis; L5 I L5 incidence; LSA Lumbosacral angle; SR Slippage rate; ODI Oswestry Disability Index

^a^Correlations are significant at the 0.05 level (2-tailed)

## Discussion

**Fig. 2:**
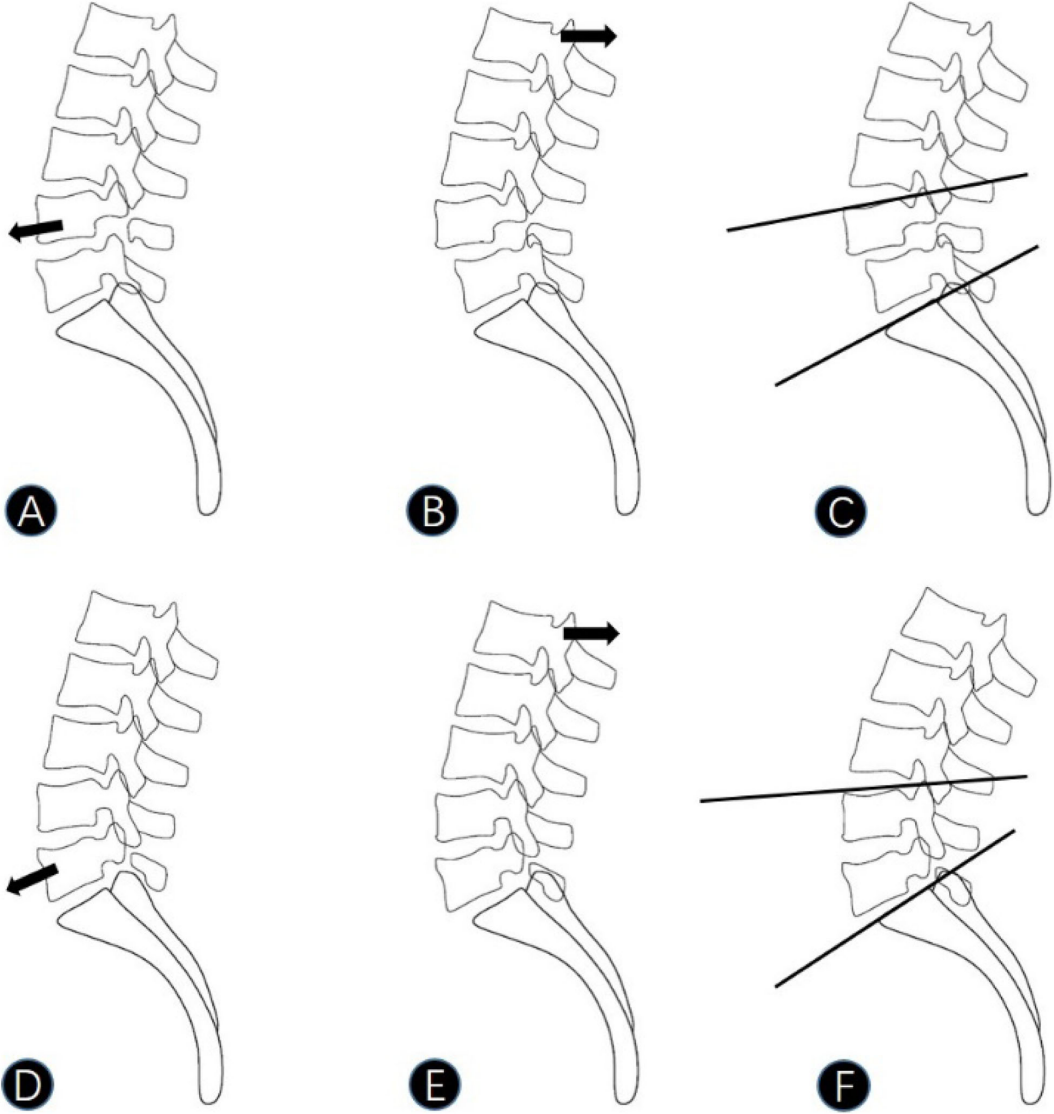
Diagram of progress of single level IS. There is a tendency of forward slippage of the vertebra with spondylolytic lesions in IS at L4 or L5 (**a, d**). With the anterior slippage of the vertebra, the segments above the spondylolisthesis hyperextend and the lordosis increases in the cranial zone of the lumbar spine to limit the excessive forward shifts of the center of gravity (**b, e**). Segmental lordosis of L4/5 in L5 IS increased while that of L5/S1 in L4 IS remained nearly unchanged. With the same degree of degeneration and lordosis of the lesion segment, LLL of the IS at L4 was smaller than that at L5 (**c, f**)

**Fig. 3:**
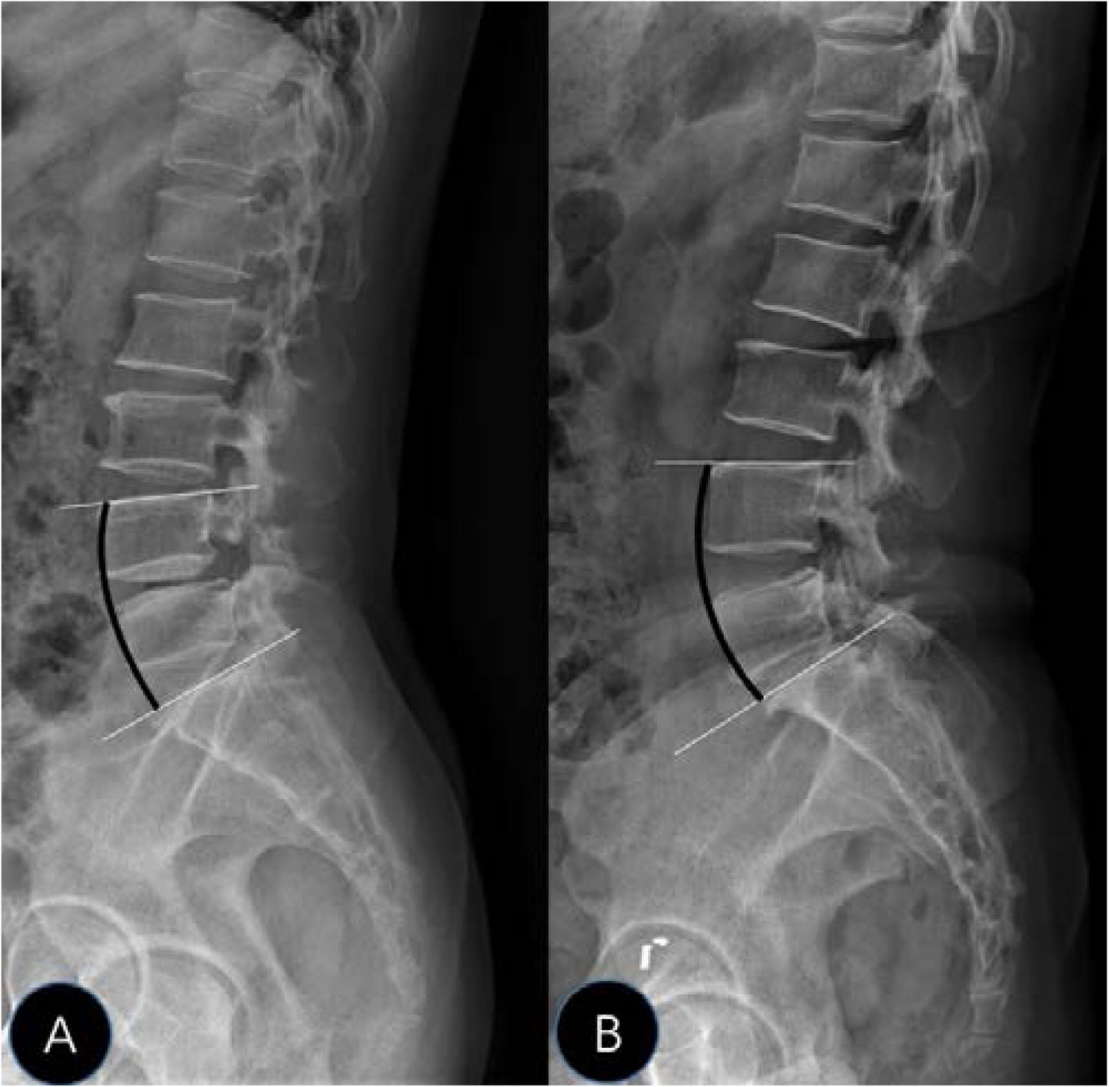
Typical changes in radiological features in IS at L4 and L5. L4 IS (**a**) showed a straighter low lumbar curvature than L5 IS (**b**)

By comparing imaging parameters between the two groups, we found that patients in the L4 IS group had smaller LLL than those in the L5 IS group (Table 1). An age-matched analysis did not reveal any significant influence of age on either group (Table 2). Hyun et al. [12] divided lumbar lordosis into three parts, of which LLL describes lordosis of the L4–S1 and counts for the two-thirds of the total lumbar lordosis [17]. As most cases of IS were located at L4–S1, LLL was included in the present study. The difference in LLL between the two groups could be explained by the special compensation mechanism of IS. For most people with complete pars interarticularis of the vertebral arch, the local sagittal imbalance of the lesion segment is always compensated by hyperextension of adjacent segments, restricting the consequence of lumbar kyphosis on the load of axis gravity [18]. However, for patients with spondylolytic lesions, the imbalance of the lesion segment always progresses to anterior slippage of the upper vertebra. With the anterior slippage of the vertebra, the segments above the spondylolisthesis hyperextend and the lordosis increases in the cranial zone of the lumbar spine, limiting the excessive forward shifts of the center of gravity [19]. This also explains the higher LL among patients with L5 IS in many studies [8–10]. As a result, segmental lordosis of L4/5 in L5 IS increased while that of L5/S1 in L4 IS remained nearly unchanged. With the same degree of degeneration and lordosis of the lesion segment, LLL of the IS at L4 was smaller than that at L5 (Fig. 2). Typical changes in radiological features of patients with single-level IS at L4 and L5 are shown in Fig. 3. We observed a straighter low lumbar curvature in the L4 IS.

Roussouly et al. [16] proposed four types of sagittal lumbo-pelvic alignment using lateral radiography. Certain types of sagittal lumbo-pelvic alignment were more frequently associated with specific degenerative diseases [20]. Patients with symptomatic disc herniation were most commonly classified as Type 1 or 2, and patients with spinal stenosis as Type 4 [16]. Funao et al. [21] found that degenerative spondylolisthesis tended to be classified as Type 3 or 4. In our study, most patients with single-level IS were classified as Type 3 or 4 (Table 3). However, the most frequent type was Type 2 in the L4 IS group and Type 3 in the L5 IS group. As Roussouly et al. [16] pointed out, Type 2 patients had flatter low lumbar curvature than Type 3 patients. Different distributions of Roussouly type among patients with L4 IS and L5 IS also suggested a straighter low lumbar curvature among patients with IS at L4.

In the present study, patients with IS at L4 were found to be older than those with IS at L5. We believe that this could be related to the biomechanics of the lumbar spine. To maintain lumbar lordosis, the lower arc of the lumbar spine, mainly L4 and L5, must be tilted downward [18]. This would explain the anterior instability with sliding-producing spondylolisthesis that always occurs at L4 and L5. As L5 is located at the lumbosacral junction with greater downward tilt, the shear force at L5 and its posterior structure is greater than that at L4. A smaller anterior shear force at L4 and its posterior structure results in later fracture of the bilateral pars interarticularis at L4, which is likely why we observed a higher proportion of older patients in the L4 IS group. However, DDG, SR, and ODI showed no significant differences in our study, even when accounting for age. This result was discordant with the conclusions and speculations of previous anatomical studies [6]. The disagreement was caused mainly by difference in study subjects; the subjects in our study were in urgent need of interventions while those in previous anatomical studies had no clinical symptoms or only mild symptoms. Many studies have shown that L4/5 is more unstable than L5/S1 due to lack or less of connections between iliolumbar ligament and L4 [5, 22]. Despite the similar severity of clinical symptoms and slippage, we believe that patients with IS at L4 deserve more attention.

Comparisons between Tables 4 and 5, reveal that fewer sagittal lumbo-pelvic parameters were significantly correlated to SR in the L4 IS group than the L5 IS group. There were fewer significant relationships among sagittal lumbo-pelvic parameters in the L4 IS group. This could be due to the discontinuity of the lumbar curve in IS at L4. PI, first proposed by Duval-Beaupere [23], has now been proven to be correlated to the grade and progression of slippage in IS as a basic anatomical parameter. However, these studies only focused on IS at L5 or simply ignored different lesion segments [8–10]. Oh et al. [24] pointed out that PI could be a progressive factor for slippage in IS at L5, but not at L4, and concluded that for patients with IS at L4, segmental instability and disc degeneration in L4/5 could have a greater influence on the pathological mechanism of slippage. In the current study, we also observed that PI was positively related to SR in the L5 IS group but not in the L4 IS group. PI was also significantly correlated with all other sagittal lumbo-pelvic parameters in the L5 IS group, but not in the L4 IS group. Thus, PI is an important parameter to be considered in adults with IS at L5.

In terms of the relationship between sagittal lumbo-pelvic alignment and SR, we found that only L5 I showed a significantly positive correlation with SR in both groups. L5 I, first introduced by Roussouly, was often used as a sagittal lumbo-pelvic parameter to assess spinopelvic morphology in high-grade spondylolisthesis and for surgical follow-up, especially when there was sacral doming [13]. Although there were significant positive correlations between L5 I and SR in both groups, the bases of the correlations were different. For L5 IS, this positive correlation was mainly due to the secondary change caused by slippage of L5. With the anterior slippage of L5, the upper end plate of L5 gradually inclines downward and forward, and the vertical distance between L5 and the hip joint decreases, thus increasing L5 I [25]. But for IS at L4, the structure under L4/5 is relatively stable. L5 I is more similar to a constant like PI in IS at L5, which can be considered a progressive factor for slippage in IS at L4. PI is an effective biomechanical parameter for predicting progression of spondylolisthesis [26]. For patients with IS at L5 and high PI, close follow-ups and early conservative treatment are very important. Early surgery may be beneficial. As for patients with IS at L4, high L5 I is worth examining as it contributes a certain reference value like PI in IS at L5 for clinical doctors when making treatment decisions.

The sagittal lumbo-pelvic alignment was not only closely related to SR but also the degree of clinical symptoms of patients with spondylolisthesis. Tanguay et al. [27], through analysis of 96 patients with L5 spondylolisthesis, proposed that decreased LSA is significantly correlated with a decline in the physical aspect of quality of life. Wang et al. [28] showed that PT, SS, and LL had significant correlations with ODI in patients with severe L5 IS. In our study, only SS and L5I showed a significant correlation with ODI in the L5 IS group, while no sagittal lumbo-pelvic parameters were significantly correlated with ODI in the L4 IS group.

Contrary to our expectations, we found no significant correlations between SR and ODI in either group. This revealed that there were no associations between the severity of clinical symptoms and degree of slippage. Considering that most patients in our study had mild to moderate degree of slippage (SR < 50%), it was reasonable to conclude that the severity of clinical symptoms of patients with low-grade IS was more closely related to their tolerance of lumbar spinal stenosis and nerve root compression rather than to the grading of images.

There were some limitations to this study. First, as a retrospective and cross-sectional study, lack of follow-up information impeded our comparison of prognosis between the two groups. However, this did provide a starting point for prospective and longitudinal studies. Second, the subjects were limited to patients who were obviously symptomatic and needed interventions, which affected the comparison results to some extent. Nevertheless, we believe that our study is of significant interest, as we are the first to discover a difference in sagittal lumbo-pelvic alignment between single-level IS at L4 and L5 and identify the reason for the difference. Our correlation analysis between SR and sagittal lumbo-pelvic parameters in IS at L4 and L5 will also be helpful in outlining appropriate interventions for patients with IS.

## Conclusions

When compared with patients with IS at L5, patients with IS at L4 were of older age and had straighter low lumbar curvature when they displayed obviously symptoms. PI was an important parameter for patients with L5 IS while for those with L4 IS, L5I deserved more attention for its significantly positive correlation with the degree of slippage.

## Data Availability

The datasets used and analyzed during the current study are available from the corresponding author on reasonable request.

## Abbreviations

IS: Isthmic Spondylolisthesis
BMI: Body mass index
PI: Pelvic incidence
PT: Pelvic tilt
SS: Sacral slope
LL: Lumbar lordosis
LLL: Lower lumbar lordosis
L5 I: L5 incidence
LSA: Lumbosacral angle
SR: Slippage rate
DDG: Disc degeneration grade
ODI: Oswestry Disability Index
MRI: magnetic resonance imaging
